# A single-center randomized, controlled trial investigating the efficacy of a mHealth ECG technology intervention to improve the detection of atrial fibrillation: the iHEART study protocol

**DOI:** 10.1186/s12872-016-0327-y

**Published:** 2016-07-16

**Authors:** Kathleen T. Hickey, Nicole R. Hauser, Laura E. Valente, Teresa C. Riga, Ashton P. Frulla, Ruth Masterson Creber, William Whang, Hasan Garan, Haomiao Jia, Robert R. Sciacca, Daniel Y. Wang

**Affiliations:** Columbia University School of Nursing, 622 W. 168th St., New York, NY 10032 USA; Columbia University Medical Center, 630 W. 168th St., New York, NY 10032 USA

**Keywords:** Atrial fibrillation, Electrocardiogram, Smartphone, Quality of life, Mobile health, Self-management, Quality-adjusted life years

## Abstract

**Background:**

Atrial fibrillation is a major public health problem and is the most common cardiac arrhythmia, affecting an estimated 2.7 million Americans. The true prevalence of atrial fibrillation is likely underestimated because episodes are often sporadic; therefore, it is challenging to detect and record an occurrence in a “real world” setting. To date, mobile health tools that promote earlier detection and treatment of atrial fibrillation and improvement in self-management behaviors and knowledge have not been evaluated. This study will be the first to address the epidemic problem of atrial fibrillation with a novel approach utilizing advancements in mobile health electrocardiogram technology to empower patients to actively engage in their healthcare and to evaluate impact on quality of life and quality-adjusted life years. Furthermore, sending a daily electrocardiogram transmission, coupled with receiving educational and motivational text messages aimed at promoting self-management and a healthy lifestyle may improve the management of chronic cardiovascular conditions (e.g., hypertension, diabetes, heart failure, etc.). Therefore, we are currently conducting a randomized controlled trial to assess the efficacy of a mobile health intervention, iPhone® Helping Evaluate Atrial fibrillation Rhythm through Technology (iHEART) versus usual cardiac care.

**Methods:**

The iHEART study is a single center, prospective, randomized controlled trial. A total of 300 participants with a recent history of atrial fibrillation will be enrolled. Participants will be randomized 1:1 to receive the iHEART intervention, receiving an iPhone® equipped with an AliveCor® Mobile ECG and accompanying Kardia application and behavioral altering motivational text messages or usual cardiac care for 6 months.

**Discussion:**

This will be the first study to investigate the utility of a mobile health intervention in a “real world” setting. We will evaluate the ability of the iHEART intervention to improve the detection and treatment of recurrent atrial fibrillation and assess the intervention's impact on improving clinical outcomes, quality of life, quality-adjusted life-years and disease-specific knowledge.

**Trial registration:**

NCT02731326; Verified April 2016

## Background

Atrial fibrillation (AF) is a major public health problem and is the most common cardiac arrhythmia, affecting an estimated 2.7 million Americans. By 2050, the number of Americans with AF will exceed 12 million [1–3]. This rise in AF is due to the advancing age of the population and the number of individuals living with multiple cardiovascular chronic conditions (e.g., heart failure, hypertension, heart disease, diabetes) that are known to be associated with the development of AF [4, 5]. The true prevalence of AF is likely underestimated because episodes are often sporadic; therefore, it is challenging to detect and record an occurrence of AF in a “real world” setting. In fact, AF recurrence is estimated to be 50 % in the first year after the initiation of treatments that restore a normal heart rhythm, highlighting the need for improved electrocardiogram (ECG) methods for detecting AF [6].

The market for mobile health (mHealth) and affordable health technology continues to rapidly increase worldwide. In fact, almost 28 % of the world’s population own and use a smartphone, and this number is expected to increase to approximately 50 % by 2018 [7]. While current clinical guidelines advocate for AF screening, the application of simple, acceptable, easy to use “real world” approaches using mobile phones for ECG monitoring that enable patients to self-manage and self-diagnose is emerging.

To date, mHealth tools that promote earlier detection and treatment of AF, adherence to cardiovascular regimens, and improvement in self-management behaviors and AF knowledge have not been evaluated. Additionally, the impact of educational text messaging on the self-management of AF-associated chronic cardiovascular conditions has not been investigated in an underserved, primarily Latino population. Prior to 2012, mHealth technology for capturing and sending ECG rhythms to healthcare providers from a smartphone for rapid real-time evaluation did not exist [8].

Utilizing recent developments of smartphone ECG technology, the Evaluate Atrial fibrillation Rhythm through Technology (iHEART) study is a randomized controlled trial (RCT) that will assess the efficacy of an mHealth intervention versus usual cardiac care. This study will be the first to address the epidemic problem of AF with a novel approach using advancements in mHealth ECG technology to empower patients to actively engage in their healthcare. Detection and treatment (e.g., medication adjustments, cardioversion) of AF may be improved through more rapid patient-provider communication facilitated by daily mHealth ECG monitoring. The impact of AF, chronic conditions, and prescribed treatments on quality of life (QoL) and quality-adjusted life-years (QALYs) will also be evaluated. Furthermore, sending daily ECG transmissions coupled with receiving educational and motivational text messages aimed at promoting self-management and a healthy lifestyle may improve co-existing chronic cardiovascular conditions (e.g., hypertension, diabetes, heart failure, etc.).

This article describes the study design and research methods used to evaluate iHEART (Clinicaltrials.gov ID: NCT02731326), a randomized controlled trial to determine whether participants receiving behavior altering motivational text messages will demonstrate a greater improvement in their cardiovascular measures (e.g., blood pressure, glucose levels) and AF knowledge from baseline to 6 months as compared to those in the usual care group. The primary hypothesis is that participants utilizing the iHEART intervention are more likely to have AF episodes detected and receive subsequent treatment for recurrent AF earlier as compared to the usual care group. The usual care group receives “guideline-directed medical care” defined by the treating cardiologist and the 2014 ACC/AHA/HRS guidelines [9]. Secondary findings will examine whether participants in the iHEART intervention will have a better self-reported QoL and improved QALYs as compared to those in the usual care group.

## Methods/Design

### Study design

**Fig. 1:**
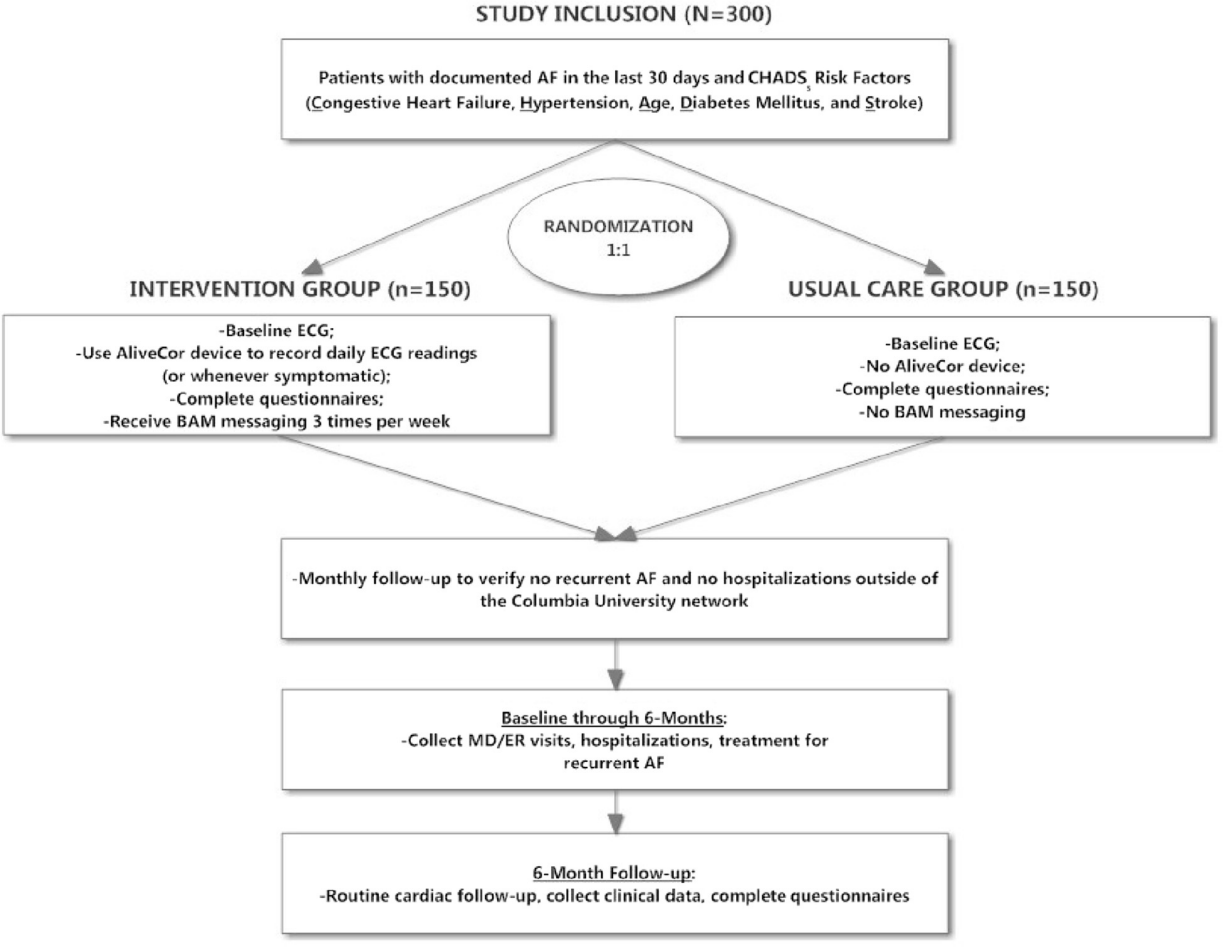
Flowchart for study procedures

The iHEART study is a single-center, prospective, RCT (Fig. 1). The primary aims of the iHEART study are to: (1) document AF using real-time ECG capture; (2) evaluate the impact on AF treatment and QALYs; and (3) evaluate the effectiveness of text messaging on AF knowledge and promoting proactive self-management of multiple chronic conditions. Participants are randomized to one of two groups: iHEART (intervention group) or usual care (control group). All participants enrolled will be followed for a period of 6 months. This study protocol adheres to the appropriate standards of reporting as stated in the Standard Protocol Items: Recommendations for Interventional Trials statement and facilitates the methodology of the Consolidated Standards of Reporting Trials statement for later dissemination of the results of the outlined RCT [10, 11].

### Study population

Patients will be screened for eligibility. Those who meet inclusion criteria will be approached for enrollment after the patient’s provider agrees to the study protocol and is willing to receive and review ECG transmissions. The details of the study will be explained in English or Spanish, and patients will be given an opportunity to ask questions before signing informed consent. A copy of the consent form will be given to the patient.

Inclusion criteria will include documented AF that has been treated in the last 30 days resulting in the restoration of normal sinus rhythm. The operational definition of AF for this study will include the presence of AF captured by the AliveCor® Mobile ECG, a standard 12 lead ECG, Holter, or any other external recording mechanism that documents AF. Over a six month period, those individuals with ≤ 7 days of AF will be considered paroxysmal, and those with greater than 7 days of continuous AF will be defined as persistent AF per the AHA/ACC/HRS guidelines [9].

The main exclusion criteria for participants include documented permanent (chronic) AF, unwillingness to use technology and have one’s clinical data collected over the study period, unwillingness to receive and read cardiovascular text messaging three times a week, and patients who have a documented medical history of cognitive impairment. The complete list of inclusion and exclusion criteria is provided in Table 1.

**Table 1:** Inclusion and exclusion criteria

Inclusion criteria	Exclusion criteria
• Males and females (English or Spanish speaking) age ≥ 18 years with a prior history of AF in the last 30 days that was treated and normal rhythm restored • Already utilizes a smartphone prior to enrollment • Ability to successfully use the AliveCor™ Heart Monitor, capture a baseline ECG, and transmit on the day of enrollment • Demonstrated ability to receive and read text messages on the day of enrollment • Willingness to complete the study questionnaires at baseline and 6 months	• Documented permanent (chronic) AF • Unwillingness to have their clinical data collected over the study period • Unwillingness to receive and read cardiovascular text messaging three times a week.

### Data and safety monitoring

The study was approved by the Columbia University Medical Center Institutional Review Board (IRB-AAAO2555). Participants provided written informed consent prior to enrollment and all data collection procedures are in accordance with the Health Information Portability and Accountability Act regulations.

### Sample size and power

A total of 300 participants with documented AF who are undergoing clinical treatment of their AF with electrical cardioversion, radiofrequency ablation and/or medical management to restore normal sinus rhythm will be enrolled. After a normal rhythm is restored, participants will be randomized to usual cardiac care (*n* = 150) or usual cardiac care plus the iHEART intervention (*n* = 150). The target sample size and power was calculated based on the difference in AF detection rates between the intervention group and the control group. From the preliminary data, there were 4 episodes of AF during the follow-up of 12 patients, or a detection rate of 0.33 vs. a detection rate of 8 % among controls. Using a Poisson regression with a two-sided test at a 95 % significance level (α = .05), 80 % power was used to detect a relative risk (RR) of less than 1.7 between the intervention group and the control group. The population will be recruited from the Columbia University Medical Center (CUMC) cardiac services and will include English and Spanish speaking participants.

### Randomization

The randomization process will be performed by a random computer number generator in a one-to-one block allocation ratio. In order to achieve a balance of applicable participant characteristics in both the intervention and control groups, participants will be stratified based on age and gender. Specifically, when the sample size is small, block randomization is commonly used in clinical trials to reduce bias and achieve balance in the allocation of participants to treatment arms. Block randomization increases the probability that each treatment arm contains an equal number of participants by sequencing individuals by block [12].

### Outcome assessments

Upon study entry, all relevant clinical data will be collected at baseline and monthly throughout six months via an electronic medical records system review. This will be used to capture and quantify cardiac outcomes in both groups. In addition, all outside medical records will be requested, and participants will be contacted monthly to capture any hospitalizations, cardiac events, or changes in therapy that may occur outside of our center and clinics. AF in the usual care group may be detected during routine follow-ups with providers, as well as during self-evaluations such as a pulse check initiated as a result of participant-reported symptoms or changes in health status. AF may also be captured by a 12-lead ECG, pulse check, or heart sound auscultation during an exam that reveals an irregular heart rhythm. Participants in the usual care group may seek medical evaluation by their providers because of a change in their health status or symptoms (shortness of breath, palpitations, fatigue), which may lead to further cardiac monitoring or testing (e.g., echocardiogram) that may reveal AF. This could include Holter monitoring (24 h) or extended cardiac monitoring (7–30 days) that may document AF and be the basis for changes in treatments prescribed by their providers (e.g., medication changes or dosage adjustments, cardioversion). In the intervention group, AliveCor® Mobile ECG transmissions will be read daily to detect recurrence of AF or other arrhythmias. All results and any treatment changes due to mobile ECG findings will be recorded over the course of the study period.

All study participants will be asked to complete a series of questionnaires including the Atrial Fibrillation Knowledge Scale (AFKS), the Canadian Cardiovascular Society Severity in Atrial Fibrillation scale (CCS-SAF), the Atrial Fibrillation Effect on Quality of Life (AFEQT), the Control Attitudes Scale-Revised (CAS-R), the Morisky 4-item Self-Report Measure of Medication-Taking Behavior (MMAS-4), the Self-Efficacy for Appropriate Medication Use Scale (SEAMS), the Short Form Health Survey (SF-36 Quality of Life), European Questionnaire 5 Dimensions (EQ-5D), the Patient Health Questionnaire (PHQ-9), and the State Trait Anxiety Inventory (STAI). These measures will be administered to both the intervention and control groups at baseline prior to any procedures, such as a cardioversion or ablation, and again at the 6-month follow-up visit for the purpose of comparison. All questionnaires have been used in similar cardiac populations and those diagnosed with/at risk for the development of AF.

### Study intervention

Participants randomized to the iHEART intervention will receive an iPhone® and cellular service plan with unlimited data/text messaging, the AliveCor® Mobile ECG, and Behavioral Altering Motivational (BAM) text messaging three times a week for six months. BAM is an in-house database that automatically sends one-way text messages to the intervention participant related to their AF, as well as two underlying cardiovascular risk factors of their choosing.

#### AliveCor™ heart monitor

**Fig. 2:**
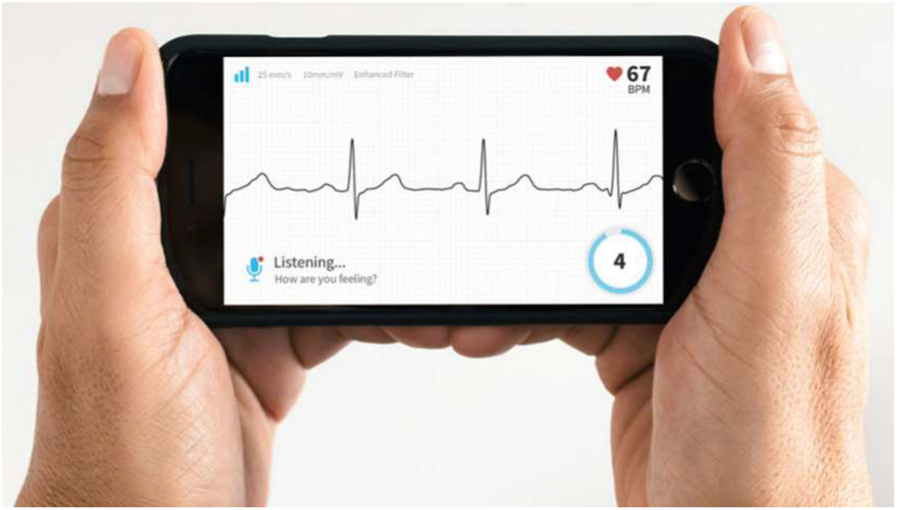
AliveCor™ Heart Monitor attached to an iPhone®

The AliveCor® Mobile ECG, an FDA-approved smartphone technology, works through a free application, “Kardia.” It captures a highly sensitive (98 %), specific (97 %), and accurate (97 %) single-lead ECG recording through two electrodes on the back of the smartphone [8]. When the application is opened, recording begins automatically when both electrodes make contact with skin (Fig. 2). Transmissions are automatically uploaded to the study portal within the AliveCor® “cloud”. All participants will receive in-person training on the use of the study equipment at enrollment. A return demonstration from the participant to the research coordinator will be required at baseline enrollment. This will include how to capture an ECG daily and in the setting of symptoms, as well as how to record any associated symptoms in the application.

All ECG images and any associated symptoms will be sent via WiFi or cellular network transmission to the AliveCor® cloud. A login will occur daily to the password-protected study database that will be stored on the AliveCor® cloud in order to review and interpret all ECG strips transmitted during the previous 24 h. Any clinically significant arrhythmias, including AF or other arrhythmias, detected during the daily review of ECGs over the 6-month period will be immediately sent to the provider caring for the participant. The study team will ensure the provider has received the transmission, is aware of any ECG abnormalities, and will then be responsible for communicating such findings.

#### Smartphone

The study is innovative in its use of mHealth and widely available smartphone technology, which allows rapid two-way communication between patients and healthcare providers, rather than waiting for office or clinic visits that delay care. An FDA-approved smartphone technology is being used, the AliveCor® Mobile ECG to capture and send ECG data via WiFi or cellular network for immediate interpretation. Transmission of a normal ECG while a patient is experiencing a symptom with multiple underlying diagnoses impacts immediate triage and treatment of the symptom. The AliveCor® technology allows clinicians to correlate symptoms with heart rhythms.

Unlike previous ECG technology, the AliveCor® Mobile ECG is not worn, so it eliminates the need for adhesive ECG electrodes and skin patches placed on the chest and does not require complicated changing of leads by the subject. This is a significant improvement from previously available technology. The use of tailored cardiac BAM text messaging sent to an individual’s smartphone to improve AF awareness and knowledge, as well as self-management of pre-existing chronic cardiovascular conditions associated with AF will be simultaneously evaluated.

#### Behavior Altering Motivational (BAM) text messaging

BAM one-way text messages are sent to the intervention participant three times per week and pertain to their AF, as well as two underlying cardiovascular risk factors of the patient’s choosing (weight management, physical activity, hypertension, heart failure, diabetes, etc.). Starting on the day of enrollment, intervention participants will receive text messages every Wednesday related to AF management, as well as a message on Monday related to risk factor one and a message on Friday related to risk factor two. BAM text messages will be systematically selected from a bank of text messages developed through collaboration by the study team and an expert interdisciplinary panel from the American Heart Association. All messages are available in English and Spanish according to participant preference.

### Control group (usual care)

Participants randomized to usual care will continue their guideline-directed clinical management and follow-up as determined by their providers’ interpretation of the 2014 ACC/AHA/HRS Guideline for the Management of Patients with Atrial Fibrillation [9].

### Study end-points

The primary endpoint is detection of recurrence of AF. Secondary endpoints include treatment changes as a result of early detection of AF recurrence, study questionnaire scores at baseline and six months, including QoL, QALYs, and improvement in cardiovascular measures (e.g., blood pressure, glucose levels) and AF knowledge from baseline to 6 months. Outcome assessors will be blinded to group allocation.

### Statistical analysis

We will define and calculate recurrent AF detection rate as the ratio of the number of recurrent AF episodes to the number of person-months of follow-ups. We will compare the difference in recurrent AF detection rate over the six-month study period between the control group and the iHEART intervention group. Analysis of the potential moderating effects of demographic factors (e.g., age, gender, etc.) as well as the difference between the groups will be performed using multivariate Poisson regression.

We will compare the time-to-treatment between the iHEART intervention group and the control group with the application of survival analysis methods. For those who are treated for recurrent AF, the time-to-treatment is the time from beginning of study to the time of treatment. Those who are not treated for recurrent AF will be censored at the end of the sixth month, or the time of death should a subject die before the sixth month. We will apply the Cox proportional hazard model to examine and test the difference in the time-to-treatment between the intervention group and control group, with the adjustment for other potential confounders (e.g., age, gender). QALYs are calculated as follows: for each month of life lived at an EQ-5D index of x, the QALYs will be x/12 = 0.0833x years. We will calculate QALYs during the 6-month study period for all participants by summarizing QALYs from baseline through sixth month. We will compare QALYs between the iHEART intervention group and the control group using a two-tailed independent sample *t*-test. To adjust for potential confounders (e.g., age, gender), multivariate linear regression models will be used. Additionally, we will compare change of QoL and symptoms of AF scores (AFEQT, AFKS, EQ5D and CCS-SAF scales, etc.) from baseline to 6 months between the two groups and between those with and without AF using linear mixed models (growth model).

## Discussion

This article describes the development and implementation of iHEART, a randomized controlled trial focusing on the potential for more timely detection and treatment of AF by actively engaging patients in their cardiac care. Occurrence rates of AF after treatment with an electrical or pharmacological cardioversion and/or radiofrequency ablation to restore a normal sinus rhythm remain high and can result in serious adverse cardiac outcomes if AF remains undetected and untreated. In fact, this RCT responds directly to a call to explore mHealth as a tool for stroke prevention secondary to paroxysmal AF [13].

This protocol aims to capitalize on the AliveCor® Mobile ECG’s documented ability to detect recurrent AF by forwarding abnormal ECG transmissions to the participant’s healthcare practitioner in real-time. Any resulting changes in clinical management, such as medication adjustments or procedures (i.e., repeat cardioversions, ablations, etc.), will be captured and compared with the mechanism for detection of AF and the clinical management in the usual care group. Our trial is currently recruiting patients whose AF has been previously treated with ablation, as well as any other intervention including electrical and pharmaceutical cardioversion with a documented return to normal sinus rhythm. Participants transmit ECGs daily, and more frequently if they are experiencing cardiac symptoms that may indicate AF, leading to an unprecedented number of data points. We anticipate approximately 30,000 ECG transmissions from the intervention arm.

Unique to this protocol is the exploration of the impact of text messaging on participant knowledge of AF and behavior modification of associated cardiac risk factors, such as hypertension, diabetes and heart failure. The iHEART text messages were developed in conjunction with an expert interdisciplinary panel from the American Heart Association and are available to participants in English or Spanish. The utility of text messaging in reducing or preventing illness through self-management and behavior alteration may reduce the burden of AF and improve participants’ quality of life and quality-adjusted life years. Utilizing an approach where individuals can visually capture and see their real time ECG may prove successful in improving AF detection through self-management.

Real time ECG transmission in the setting of multiple cardiac diagnoses may effectively guide treatment in the ambulatory setting preventing unnecessary visits and associated healthcare utilization. For example, a patient experiencing symptoms with multiple underlying diagnoses who transmits a normal ECG can be ruled out for recurrent AF and instead be treated accordingly. Muhlestein and colleagues concluded the AliveCor® Mobile ECG is an adequate determinate of STEMI when compared to 12 Lead ECGs at the time of presentation [14]. Also, the AliveCor® Mobile ECG’s ability to accurately capture arrhythmias in the pediatric population has been confirmed by the SPEAR Trial, [15] as well as Ferdman et al. who used AliveCor® Mobile ECG to diagnose cases of pediatric supraventricular tachycardia [16]. While Muhlestein and colleagues did successfully monitor the QTc interval for AF patients treated with dofetilide using the AliveCor® Mobile ECG, [17] and Tarakji’s iTRANSMIT study [18] concluded the AliveCor® Mobile ECG was an accurate alternative for detection of AF recurrence after ablation among 60 participants, the evaluation of the usefulness of the AliveCor® Mobile ECG in combination with the Kardia application in the setting of AF has been limited thus far. The silent nature of AF often leads to a gross underdetection of recurrence [19]. Our study’s innovative design and implementation of this technology has the potential to address this issue among urban communities.

Bloss et al. found no difference in overall healthcare utilization over a 6 month period among persons with AF utilizing the AliveCor® application, however, their AF arm required participants to transmit an ECG only when symptomatic resulting in a mean of 57 transmissions per participant [20]. Their study did not include a mechanism for capturing silent episodes of AF, unlike our RCT, which requires daily transmissions regardless of symptoms.

## Conclusions

If the aims of this study are achieved, we believe this may alter existing guidelines for how ECG monitoring and patient education are approached, as well as lay the foundation for using mHealth interventions aimed at improving health promotion, QoL and disease prevention. The potential application of this project extends to using mHealth for ECG monitoring to other underserved, geographically diverse populations, as well as in other fields of personalized healthcare. The utility of mHealth in public health programs aimed at preventing illness through self-management of behavior and treatment of other chronic diseases known to be associated with AF, such as heart failure, may improve healthcare outcomes in the future.

### Trial status

The study protocol and amendments were approved by the Columbia University Human Research Protection Office prior to the start of the study (IRB-AAAO2555). The trial is also registered at www.clinicaltrials.gov (NCT02731326). The study is currently ongoing, having recently entered its second year of enrollment.

## Abbreviations

AF, atrial fibrillation; AFEQT, atrial fibrillation effect on quality of life; AFKS, atrial fibrillation knowledge scale; BAM, behavioral altering motivational messaging; CAS-R, control attitudes scale-revised; CCC-SAF, Canadian Cardiovascular Society Severity in Atrial Fibrillation Scale; CONSORT, consolidated standards of reporting trials; CUMC, Columbia University Medical Center; ECG, electrocardiogram; EQ-5D, European Questionnaire 5 Dimensions; iHEART, iPhone® Helping Evaluate Atrial fibrillation Rhythm through Technology; mHealth, mobile health; MMAS-4, Morisky 4-item self-report measure of medication-taking behavior; PHQ-9, patient health questionnaire; QALYs, quality-adjusted life-years; QoL, quality of life; RCT, randomized controlled trial; SEAMS, Self-Efficacy for Appropriate Medication Use Scale; SF-36, short form health survey; STAI, state trait anxiety inventory
